# *EGFR* 20外显子插入突变非小细胞肺癌规范化诊疗中国专家共识（2023版）

**DOI:** 10.3779/j.issn.1009-3419.2023.106.10

**Published:** 2023-05-20

**Authors:** 中国临床肿瘤学会非小细胞肺癌专家委员会

**Keywords:** 肺肿瘤, EGFR 20外显子插入突变, 共识, Lung neoplasms, Epidermal growth factor receptor exon 20 insertion mutations, Consensus

## Abstract

随着非小细胞肺癌（non-small cell lung cancer, NSCLC）精准诊疗的不断发展，表皮生长因子受体（epidermal growth factor receptor, EGFR）20外显子插入（exon 20 insertion, ex20ins）突变作为罕见突变亚型逐渐被关注，其异质性高，不同插入位点亚型临床获益不同，预后极差，对现有传统治疗方案疗效有限，且常规聚合酶链反应（polymerase chain reaction, PCR）漏检率近50%，因此，在临床诊疗过程中更应该引起高度重视。本共识专家组通过国内外文献及临床数据的参考，并且结合专家自身临床经验，形成EGFR ex20ins突变NSCLC临床规范化诊疗专家共识，分别从疾病认知、疾病检测、疾病治疗和疾病相关新型靶向药物研发现状等方面提出共识性建议，以期为各级临床医师提供用药参考。

## Abstract (en)

With the development of precision diagnosis and treatment for non-small cell lung cancer (NSCLC), the epidermal growth factor receptor (EGFR) exon 20 insertion (ex20ins) mutations, as a rare subset of EGFR mutaions, have gradually attracted attention recently. The heterogeneity of EGFR ex20ins mutations is very high, different variants have different clinical benefits, and the prognosis is extremely poor. The available traditional treatment outcomes are poor in patients with EGFR ex20ins positive NSCLC and polymerase chain reaction (PCR) tests would miss aprocimately 50% of the variants. Therefore, high attention should be paid to EGFR ex20ins positive NSCLC during the clinical practice. The expert panel has formed a consensus on the standardized clinical diagnosis and treatment of EGFR ex20ins mutation NSCLC through reference to literature and clinical data, and combined with the experts' own clinical experience, the consensus recommendations including clinicopathologic characteristics, therapies, testing methods and recent relevant clinical trials for NSCLC patients with EGFR ex20ins mutation, in order to provide medication reference for clinical physicians at all levels.

## 前言

非小细胞肺癌（non-small cell lung cancer, NSCLC）约占所有肺癌的85%，是肺癌主要病理组织学类型^[1]^，我国最常见的肺癌驱动基因是表皮生长因子受体（epidermal growth factor receptor, EGFR）（约占非鳞NSCLC的50%），伴有EGFR常见突变的NSCLC患者靶向治疗的疗效与分子分型的关系已经在临床实践中得到证实^[2]^。EGFR 20外显子插入（exon 20 insertion, ex20ins）突变是一类EGFR突变的亚型，是继EGFR 19外显子缺失（19-Del）和21外显子L858R点突变（21-L858R）两大常见突变外EGFR的第三大突变^[3]^。随着临床越来越多化合物的探索及新型靶向药物的获批，该罕见靶点逐渐被关注。首个针对该类患者的口服靶向药物琥珀酸莫博赛替尼刚刚在中国获批，开启了中国EGFR ex20ins突变NSCLC靶向治疗的新纪元。但在临床实践中，临床医生对EGFR ex20ins突变NSCLC的疾病认知、如何精准选择患者、如何进行规范化诊断、治疗及管理不良反应（adverse events, AEs）等临床标准有待进一步完善。因此，由中国临床肿瘤学会（Chinese Society of Clinical Oncology, CSCO）NSCLC专家委员会牵头，组织肺癌领域专家，通过参考国内外文献及临床数据，并且结合专家自身临床经验，形成EGFR ex20ins突变NSCLC临床规范化诊疗中国专家共识，以期为各级临床医师提供用药参考。

**专家共识1：EGFR ex20ins突变是EGFR的第三大突变，约占EGFR突变NSCLC患者的12%，其恶性程度高，异质性强（亚型达100多种），且不同插入位点亚型临床获益不同，患者预后差，临床需引起重视。**

EGFR基因位于7p12染色体上，由28个外显子和27个内含子组成。中国NSCLC人群中，EGFR突变比例为50%^[4]^。其中常见突变为EGFR 19外显子缺失和21外显子L858R点突变，约占EGFR突变的85%，EGFR酪氨酸激酶抑制剂（EGFR-tyrosine kinase inhibitors, EGFR-TKIs）治疗可为这类常见EGFR突变NSCLC患者带来显著的临床获益^[5⇓-7]^。

EGFR ex20ins突变是除EGFR两种常见突变（19外显子缺失和21外显子L858点突变）以外的第三大突变，于2004年首次被提出^[8,9]^，其占所有NSCLC腺癌突变的2.3%左右，约占EGFR突变NSCLC患者的12%^[10⇓⇓-13]^。根据中国大样本回顾性研究^[13⇓-15]^数据，EGFR ex20ins突变占中国肺癌患者总数的2.1%-2.27%，就发病率而言，东西方人群尚未发现有显著差异。临床特征与EGFR常见突变类似，EGFR ex20ins突变多见于不吸烟、女性、亚裔和肺腺癌患者^[16,17]^。

EGFR ex20ins突变（图1）的特征为以α-C-螺旋附近密码子761与775之间框内插入和/或复制，致EGFR通路激活^[18,19]^。从插入位点与氨基酸序列角度看，EGFR ex20ins突变异质性强，其亚型达100多种，约10%位于α-C-螺旋的C末端（761-766），约90%位于α-C-螺旋后的Loop环结构域。EGFR ex20ins Loop环结构域内插入突变又可分为Loop环近端（767-772）（占EGFR ex20ins突变的66%-72%）与Loop环远端（773-775）（占EGFR ex20ins突变的27%-28%）^[20]^。前者代表性突变包括S768dupSVDA767dupASV、D770insNPG以及D770delinsGY，后者代表性突变包括H773insNPH、H773dupH、V774insAV以及V774insPR。中国真实世界研究数据（165例EGFR ex20ins突变）^[21,22]^显示V769_D770insASV为最常见的突变亚型，占23.0%。

**图1:**
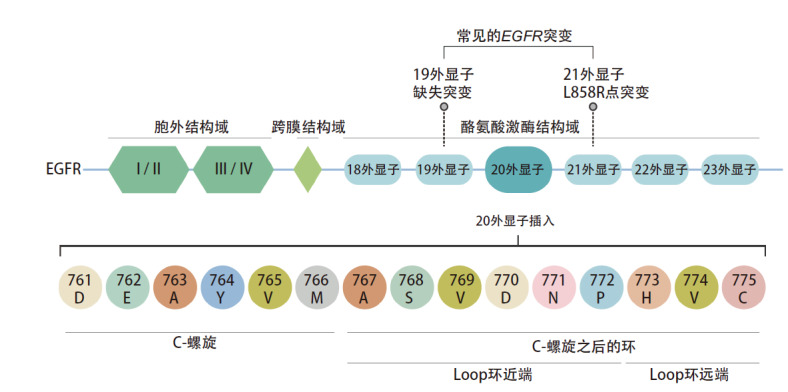
EGFR ex20ins突变示意图 EGFR：表皮生长因子受体；ex20ins：20外显子插入。

EGFR ex20ins突变的NSCLC患者中，传统的EGFR-TKIs仅对极少数（约占EGFR ex20ins突变的5.1%）的亚型有效^[23]^，例如A763_Y764insFQEA和A763_Y764insLQEA，而对其他大部分的亚型原发性耐药，疗效不佳。针对EGFR ex20ins突变的新药研发充满挑战，首先EGFR ex20ins突变会诱导EGFR的α-C-螺旋和磷酸结合环转移至药物结合口袋，形成明显的空间位阻，导致药物结合口袋缩小，进而导致传统的EGFR-TKIs结合受阻^[24]^。其次，EGFR ex20ins突变同野生型EGFR结构和三磷酸腺苷（adenosine triphosphate, ATP）的亲和力方面高度相似，该结构上的相似性使得对正常组织的毒性成为药物研发中一个令人担忧的问题^[18]^。另外，插入位点结构域的不同也影响EGFR-TKIs对EGFR ex20ins突变NSCLC的治疗效果，传统EGFR-TKIs对极少位于α-C-螺旋内的突变亚型或有一定疗效，而对位于Loop环区内的突变亚型则疗效较差^[25⇓-27]^。此外，一项来自Flatiron数据库的回顾性真实世界队列研究（2020年5月-2022年1月）^[28]^显示，与常见EGFR突变NSCLC患者相比，EGFR ex20ins突变NSCLC患者的真实世界无进展生存期（real world progression-free survival, rwPFS）仅为2.9个月（vs 10.5个月，P<0.0001），真实世界总生存期（real world overall survival, rwOS）为16.2个月（vs 25.5个月，P<0.0001），EGFR ex20ins突变NSCLC患者的预后较EGFR常见突变患者更差。

**专家共识2：在NSCLC的个体化精准治疗模式下，不同基因检测方法对EGFR ex20ins突变检出率存在明显差异，聚合酶链反应（polymerase chain reaction, PCR）检测对EGFR ex20ins突变亚型的漏检率高达50%，二代测序（next-generation sequencing, NGS）几乎能够全面覆盖EGFR ex20ins突变的不同亚型，推荐优先采用NGS作为该基因的检测手段，同时需积极推动上下级医院联动、院内临理互动，共同推进精准检测落地。**

与PCR相比，NGS可一次性、在特定时间（约10个工作日）产生覆盖基因组特定区域（从数个基因到数百个基因以致全外显子组或全基因组）的高通量测序数据，可以检测低至0.1%的等位基因突变频率^[29]^。几乎可全面覆盖NSCLC EGFR ex20ins突变的不同亚型，敏感性更高，特异性更强，在临床诊疗中有更重要的应用价值^[30]^。而PCR只能对已知的突变亚型进行检测，且仅检测低至1%的等位基因突变频率^[31]^，易漏检^[30]^，在EGFR ex20ins等分子异质性突变检测方面能力不足^[32]^。NGS及当前不同PCR的EGFR ex20ins检测覆盖率总结见表1。但与PCR相比，NGS医保覆盖率略低，费用略高，报告耗时略长。中国NSCLC患者数量庞大，更需要全面精准的诊断手段，同时应兼顾各级医院检测手段的可及性，推荐有条件的上级医院优先采用NGS作为检测手段，下级医院二线治疗前应进行NGS检测，首选组织标本检测。同时推动上下级医院联动、院内临理互动，共同推进NGS检测落地，以提高EGFR ex20ins等罕见突变的检出率。

**表1:** NGS和PCR的EGFR ex20ins检测覆盖率

检测 手段	检测公司	可检测到的EGFR ex20ins	具体亚型 (占所有突变的比例)	EGFR ex20ins 检测覆盖率 (%)
PCR	Cobas	5	V769_D770insASV or 767_769 duplicate (≈17%); D770 N77linsSVD (≈22%); D770_N771insG (≈2%); H773_V774insH (≈7%)	48
	AmoyDx 9 in 1	15	H773_V774insH; D770_N771insG; V769_D770insASV; D770_N771insVD; V769_D770inASV; H773_V774insNPH; H773_V774insQ; N771_P772insT; N771_P772insH; P772_H773insQ; H773_V774insY; V769_D770insGsV; D770_N77linG; D770_N771insG; P772_H773insDNP	58
	和实	3	V769_D770insASV (≈17%); D770_N771insG (≈2%); H773_V774insH (≈7%)	26
	透景	2	V769-D770insASV (≈17%); D770_N771insG (≈2%)	19
	雅康博	可检测到	NA	NA
	飞朔	可检测到	NA	NA
NGS	燃石 华大 艾德	几乎所有亚型		≈100

PCR：聚合酶链反应；NGS：二代测序；NA：不适用。

**专家共识3：因目前国内尚无一线治疗局部晚期或转移性EGFR ex20ins突变NSCLC靶向药物获批，建议参考无驱动基因晚期NSCLC的一线治疗。针对无法耐受或拒绝化疗/免疫治疗或体力状况（performance status, PS）评分较差等特殊的患者，一线可选择莫博赛替尼。**

目前国内尚无针对EGFR ex20ins突变NSCLC一线治疗的靶向药物获批，当前建议参考无驱动基因的局部晚期或转移性NSCLC的一线治疗，治疗原则可参考CSCO《非小细胞肺癌诊疗指南2023》^[33]^（图2）。非鳞癌NSCLC（PS=0分-1分）I级推荐：（1）培美曲塞联合铂类+培美曲塞单药维持治疗；（2）贝伐珠单抗联合含铂双药化疗+贝伐珠单抗维持治疗；（3）含顺铂或卡铂双药方案；（4）阿替利珠单抗[限程序性细胞死亡配体1（programmed death-ligand 1, PD-L1）肿瘤细胞阳性比例分数（tumor proportion score, TPS）≥50%或免疫细胞阳性比例分数（immune proportion score, IPS）≥10%]；（5）帕博利珠单抗[限PD-L1 TPS≥50%（1A类直接证据），PD-L1 TPS为1%-49%（2A类直接证据）]；（6）培美曲塞+铂类联合帕博利珠单抗或卡瑞利珠单抗或信迪利单抗或替雷利珠单抗或阿替利珠单抗或舒格利单抗或特瑞普利单抗。非鳞癌NSCLC（PS=2分）I级推荐单药化疗（吉西他滨或紫杉醇或长春瑞滨或多西他赛或培美曲塞）。鳞癌（PS=0分-1分）I级推荐：（1）含顺铂或卡铂双药方案；（2）含奈达铂双药方案；（3）阿替利珠单抗（限PD-L1 TPS≥50%或IPS≥10%）；（4）帕博利珠单抗[限PD-L1 TPS≥50%（1A类直接证据），PD-L1 TPS为1%-49%（2A类直接证据）]；（5）紫杉醇/白蛋白紫杉醇+铂类联合帕博利珠单抗或替雷利珠单抗；（6）紫杉醇+卡铂联合卡瑞利珠单抗或舒格利单抗或派安普利单抗；（7）吉西他滨+铂类联合信迪利单抗；（8）白蛋白紫杉醇+铂类联合斯鲁利单抗。鳞癌（PS=2分）I级推荐单药化疗（吉西他滨或紫杉醇或长春瑞滨或多西他赛）。

**图2:**
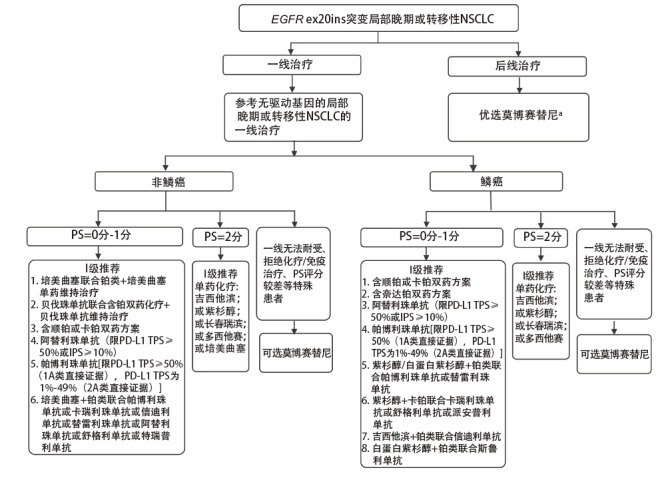
EGFR ex20ins突变局部晚期或转移性NSCLC治疗路径图 NSCLC：非小细胞肺癌；PS：体能状态；PD-L1：程序性细胞死亡配体1；TPS：肿瘤细胞阳性比例分数；IPS：免疫细胞阳性比例分数。^a^莫博赛替尼推荐剂量为160 mg ，每日一次，每天同一时间服用，可与或不与食物同服。

但传统治疗方案预后相对差（表2），EGFR ex20ins突变NSCLC患者存在未被满足的临床治疗需求。美国癌症电子病历数据库Flatiron Health截至2020年2月数据^[34]^显示，EGFR ex20ins突变NSCLC最常用的一线治疗方案为化疗及化疗联合治疗，约占全部治疗手段的60%，确认的客观缓解率（confirmed objective response rate, cORR）约为20%（n=57），中位PFS为4.5个月-5.7个月；免疫治疗作为单药一线方案，cORR仅为9.1%，PFS为3.1个月（n=11）；传统EGFR-TKIs作为单药一线方案，ORR仅为2.7%，PFS为3.3个月（n=37）。加拿大真实世界研究^[35]^亦提示EGFR ex20ins突变的一线治疗患者的OS为10.52个月，低于EGFR 19外显子缺失患者（20.55个月）和21外显子L858R点突变患者（19.10个月）。中国EGFR ex20ins突变NSCLC患者真实世界研究^[21]^数据显示，化疗作为一线治疗，ORR仅为19.2%，PFS为6.4个月（n=105）；传统EGFR-TKIs作为一线治疗时，ORR为8.7%，PFS仅为2.9个月（n=23）。德国的一项研究^[36]^也显示在EGFR ex20ins突变NSCLC患者中EGFR-TKIs作为一线治疗时，PFS为3.3个月，短于化疗的5.0个月；传统EGFR-TKIs（第一、二、三代）厄洛替尼、吉非替尼、阿法替尼和奥希替尼治疗效果均不理想，PFS为0.7个月-3.7个月。一项系统综述及meta分析^[37]^探索了EGFR ex20ins突变NSCLC患者的生存结局，共纳入23项真实世界研究，结果显示传统EGFR-TKIs、化疗及免疫治疗分别作为一线治疗时，ORR分别为6.8%、25.7%和14.0%；PFS分别为3.0个月（n=174）、5.6个月（n=455）和4.3个月（n=50），均不超过6个月；除真实世界研究外，该meta分析还纳入了19项干预性研究，结果提示第三代EGFR-TKIs不论是常规剂量还是双倍剂量都未能为EGFR ex20ins突变NSCLC患者带来良好获益。以上研究报道提示化疗、免疫治疗和传统EGFR-TKIs一线治疗EGFR ex20ins突变NSCLC患者均获益有限，一线治疗仍存在极大的未被满足的需求。对于无法耐受、拒绝一线化疗/免疫治疗、PS评分较差等特殊的患者人群，可选择莫博赛替尼，但其一线国际、多中心、III期研究正在进行中，期待研究结果进一步验证一线疗效。

**表2:** 传统治疗方案应用于EGFR ex20ins突变NSCLC一线治疗的疗效

研究者	研究类型	样本量	治疗方案	mPFS (95%CI, 月)	ORR (95%CI)	mOS (95%CI, 月)
Ou SI et al.^[34]^	美国Flatiron Health数据库回顾性研究 数据截止日期2020年2月29日	41	含铂化疗	5.7 (3.0-10.9)	19.5% (8.8%-34.9%)	17.0 (10.5-33.2)
		11	免疫单药治疗	3.1 (1.1-5.2)	9.1% (0.2%-14.2%)	11.0 (1.2-NR)
		37	传统EGFR-TKIs	3.3	2.7% (0.1%-14.2%)	10.7 (3.4-22.3)
O'Sullivan DE et al.^[35]^	加拿大真实世界研究	13	含铂化疗、免疫治疗、传统EGFR-TKIs	NA	NA	10.5 (8.0-NA)
Yang G et al.^[21]^	中国真实世界研究 数据截止日期2018年12月2日	105	含铂化疗	6.4 (5.7-7.1)	19.2%	NA
		23	传统EGFR-TKIs	2.9 (1.5-4.3)	8.7%	NA
Janning M et al.^[36]^	德国回顾性研究	26	传统EGFR-TKIs	3.3 (2.2-4.4)	NA	NA
		66	化疗	5.0 (3.2-6.7)	NA	NA
		23	免疫治疗	3.2 (2.4-4.1)	NA	NA
Kwon CS et al.^[37]^	Meta分析	174	传统EGFR-TKIs	3.0 (2.0-3.8)	6.8% (3.3%-13.5%)	16.4 (11.6-19.7)
		455	化疗	5.6 (4.6-6.9)	25.7% (19.5%-33.1%)	18.3 (11.6-19.7)
		50	免疫治疗	4.3 (3.1-5.6)	14% (6.8%-26.6%)	11.2 (11.0-11.3)

mPFS：中位无进展生存期；ORR：客观缓解率；mOS：中位总生存期；TKIs：酪氨酸激酶抑制剂；NR：未达到；CI：可信区间。

**专家共识4：EGFR ex20ins突变局部晚期或转移性NSCLC患者后线治疗，优先推荐莫博赛替尼。**

莫博赛替尼（Mobocertinib, TAK-788）于2020年4月被美国食品药品监督管理局（Food and Drug Administration, FDA）授予突破性治疗药物资格。2021年9月通过美国FDA优先审评程序并加速审批。国内，莫博赛替尼2020年被国家药品监督管理局（National Medical Products Administration, NMPA）列为“突破性治疗药物”，并获准进入“优先审评”审批程序。作为1类创新药，莫博赛替尼已于2023年1月正式获得NMPA批准，成为国内首个且唯一获批用于治疗含铂化疗期间或之后进展且携带EGFR ex20ins突变的局部晚期或转移性NSCLC成人患者的靶向药物，开创了EGFR ex20ins突变NSCLC靶向治疗的新纪元。推荐剂量为160 mg，每日一次，每天同一时间服用，可与或不与食物同时服用。针对AEs的剂量调整方案推荐如下，首次减量为减至120 mg，每日一次，第二次减量为减至80 mg，每日一次，其他针对AEs调整剂量的建议可参考莫博赛替尼药品说明书^[38]^。

莫博赛替尼是一种基于吲哚嘧啶的EGFR不可逆抑制剂，采用迭代、结构导向的研发策略，是专门针对EGFR ex20ins突变进行研发的首个小分子靶向药物。其在嘧啶环上引入了异丙酯侧链结构，使其可与ATP结合口袋内的“门卫”残基相互作用，可精准识别EGFR ex20ins与野生型EGFR的细微构象差异，分别通过EGFR-Cys797/原癌基因人类表皮生长因子受体2（human epidermal growth factor receptor-2, HER2）Cys805与突变的EGFR/HER2不可逆、共价结合，这种结构设计使得莫博赛替尼对EGFR ex20ins突变具有高度亲和力、高选择性、强效抑制作用。莫博赛替尼的代谢产物（AP32914和AP32960）对野生型EGFR和EFGR突变的细胞活性与莫博赛替尼相似，使其对EGFR ex20ins抑制作用更强且更持久^[39]^。

莫博赛替尼的获批是基于一项I期/II期临床研究（NCT02716116）^[26]^。纳入既往接受过含铂化疗的EGFR ex20ins突变NSCLC患者（n=114）[即铂类经治人群（platinum-pretreated patient, PPP）包括剂量递增研究（n=6）、扩展队列（n=22）以及EXCLAIM延展队列（n=86）]进行合并分析，PPP人群每日一次口服莫博赛替尼160 mg，其中基线伴脑转移的患者占35%，既往接受过二线及以上治疗的患者占59%，有免疫治疗史的患者占43%，有EGFR-TKIs治疗史的患者占25%。主要研究终点显示（数据截止至2021年11月1日），中位随访时间为25.8个月，独立评审委员会（Independent Review Committee, IRC）评估的cORR为28%，研究者（investigator, INV）评估的cORR为35%；IRC评估的中位缓解持续时间（duration of response, DOR）为15.8个月；INV与IRC评估的cDCR[≥部分缓解（partial response, PR）+疾病稳定（stable disease, SD）≥6周]均为78%（89/114）；IRC评估的中位PFS为7.3个月，中位OS为20.2个月。研究结果显示无论插入的位置还是突变亚型频率如何，EGFR ex20ins突变亚型均观察到不同程度的缓解[ORR：ASV/SVD/NPH亚组为31.9%（15/47）vs 其他为25.0%（12/48）；Loop环近端（767-772）为28.6%（20/70） vs Loop环远端（773-775）为25.0%（6/24）]。PPP队列中，既往接受过免疫治疗或EGFR-TKIs的患者接受莫博赛替尼治疗仍有效^[40]^。此外，患者生活质量报告^[41]^显示，在既往经治的EGFR ex20ins突变NSCLC患者中，接受莫博赛替尼治疗后患者的呼吸困难、咳嗽、胸痛等肺癌症状在2个月内得到临床意义的改善，并且在治疗期间得以维持。一项间接对照研究^[42]^探索了在既往含铂化疗经治的EGFR ex20ins突变NSCLC人群中莫博赛替尼与真实世界治疗方案的疗效（EGFR-TKIs为37%、单药化疗为26%、免疫治疗为19%、双药化疗为9%、化疗联合免疫治疗为9%）差异，研究结果显示莫博赛替尼显著优于真实世界其他治疗方案，cORR为35.1% vs 真实世界数据（real world data, RWD）为0%，中位PFS为7.3个月 vs RWD为2.5个月[风险率（hazard rate, HR）=0.28，P<0.001]，中位OS为24个月 vs RWD为9.8个月（HR=0.48, P=0.018）。基于以上研究结果（表3），推荐莫博赛替尼为含铂化疗经治EGFR ex20ins NSCLC患者的优选治疗方案。

**表3:** 莫博赛替尼应用于EGFR ex20ins突变NSCLC后线治疗

研究/研究者	分期	样本量	治疗方案	mPFS (95%CI, 月)	ORR (95%CI)	mOS (95%CI, 月)
NCT02716116 PPP队列^[26]^	I期/II期	114	莫博赛替尼 数据截止2021年11月1日	IRC评估: 7.3 (5.5-9.2)	IRC评估: 28.0% (22%-37%) INV评估: 35.0% (26%-45%)	20.2 (14.6-28.8)
Christopoulos P et al.^[42]^	间接对照研究	114 vs 122	莫博赛替尼 vs 真实世界治疗方案（含传统EGFR-TKIs、化疗及免疫治疗）	7.3 (5.6-8.8) vs 2.5 (1.5-3.4) (HR=0.28, P<0.001)	35.1% (26.4%-44.6%) vs 0%	24.0 (14.6-28.8) vs 9.8 (4.3-13.3) (HR=0.48, P=0.018)

IRC：独立评审委员会；INV：研究者；HR：风险率。

**专家共识5：莫博赛替尼的AEs谱与传统EGFR-TKIs总体相似，最常见的治疗相关AEs为胃肠道和皮肤相关，多为1级或2级，可通过积极的支持治疗、剂量调整和/或停药进行管理。**

莫博赛替尼毒性谱与已知的EGFR-TKIs相似，多为轻症，整体可控可管理。I期/II期临床研究（NCT02716116）^[40]^中（n=114），数据截止至2021年11月，莫博赛替尼最常见的治疗相关AEs（treatment-related AEs, TRAEs）为腹泻（92%）、皮疹（46%）、甲沟炎（38%）和食欲减退（37%），在≥3级TRAEs中，腹泻（23%）是唯一发生率≥10%的AEs。18%的患者因AEs的发生导致暂停用药，最常见的停药原因为腹泻（4%）、恶心（2%）、呕吐（2%）和食欲减退（2%）等。27%的患者因AEs导致减量，最常见的减量原因为腹泻、恶心和皮疹等。

此外，安全性汇集（来自NCT02716116和NCT03807778，290例实体肿瘤患者，其中包含285例NSCLC患者）分析进一步反映了莫博赛替尼在160 mg、每日一次标准下的暴露情况^[38]^，最常见的AEs为腹泻（94%）和皮疹（80%）。临床研究中20%（59/290）的患者出现3级腹泻，1例（0.3%）患者出现4级腹泻。中位腹泻发作时间为5.0 d，中位消退时间为3.0 d。≥65岁的患者的3级或4级腹泻发生率为26%（30/114），高于较年轻患者（17%, 30/176）。建议进行早期依从性的腹泻管理，如处方止泻药（如洛哌丁胺）、调整饮食、充分补液（每天1 L-1.5 L等渗液体）和患者教育。指导患者常规备用止泻药（如洛哌丁胺）。首次出现不成形或松软便或排便频率高于正常时，应开始止泻治疗。莫博赛替尼引起的皮肤相关毒性大部分严重程度较低，仅3.4%（10/290）的患者出现3级皮疹，中位皮疹发作时间为9.0 d，中位消退时间为78 d，大部分患者可通过积极皮肤护理、局部使用皮质类固醇（氢化可的松等）和/或抗生素（如莫匹罗星、克林霉素等）改善症状。

在特定药物AEs中，临床研究显示，4.5%（13/290）的患者发生间质性肺疾病（interstitial lung disease, ILD）或肺部炎症（非感染性）；其中0.7%（2/290）的患者为3级事件；12%（35/290）的患者出现校正后QT间期（QT corrected, QTc）延长，4.5%（13/290）的患者出现3级QTc延长。因QTc延长，包括尖端扭转性室性心动过速，所以需要在基线和治疗期间定期监测QTc和电解质，增加有QTc延长风险因素患者的监测频率，还应避免本品和已知会延长QTc的药物合用，避免本品和强效或中效细胞色素P450 3A（cytochrome P450 3A, CYP3A）抑制剂同时给药；同时需要根据QTc延长的严重程度，暂停、减量或永久停用莫博赛替尼。

针对莫博赛替尼的AEs处理，可参考国内《EGFR TKI不良反应管理专家共识》^[43]^和莫博赛替尼药品说明书^[38]^。

总体而言，莫博赛替尼的AEs与已知的EGFR-TKIs相关AEs相似^[44]^，患者教育、早期识别、及时积极的管理以及持续的评估有助于减少AEs发生，从而最大限度地减少剂量调整，以获得更好的潜在疗效。

**专家共识6：目前有多个针对EGFR ex20ins突变NSCLC一线及后线治疗的药物/新型化合物正在开展临床研究探索，临床医生可持续关注相关循证医学证据的产出，以共同推动该靶点临床规范化治疗。**

莫博赛替尼作为局部晚期或转移性EGFR ex20ins 突变NSCLC一线研究EXCLAIM-2（NCT04129502）^[45]^正在开展，该研究是一项国际多中心、随机化、开放性、III期临床研究，旨在比较莫博赛替尼与含铂化疗（培美曲塞+顺铂或卡铂继以培美曲塞维持）一线治疗局部晚期或转移性EGFR ex20ins突变NSCLC的有效性及安全性。

除莫博赛替尼外，目前仍有多个针对EGFR ex20ins突变NSCLC一线及后线治疗的药物/新型化合物正在开展临床研究。

舒沃替尼（Sunvozertinib, DZD9008）是一款新型针对EGFR/HER2 ex20ins突变设计的选择性、不可逆EGFR-TKIs，基于奥希替尼在C-4位采用更灵活的苯氨基结构替换甲基吲哚占据ATP结合口袋，同时对EGFR常见突变、T790M突变及罕见突变亦具有抑制活性。NMPA基于其I期/II期临床研究WU KONG 1（NCT03974022）和WU KONG 2（CTR20192097）^[25,46]^，将其纳入“突破性”药物名单。舒沃替尼用于EGFR ex20ins突变NSCLC后线治疗的WU KONG 6（CTR20211009）研究^[47]^已公布截止至2022年7月31日的数据分析结果，基线特征显示，32%的患者基线伴脑转移；既往系统抗肿瘤治疗线数（范围）为一线至三线，其中50.5%的患者既往接受过二线及以上治疗，34%的患者既往接受过免疫治疗，25.8%患者既往接受过EGFR-TKIs治疗；在300 mg、每日一次的推荐剂量下，在既往含铂化疗经治EGFR ex20ins突变NSCLC患者（n=97）中，盲态独立中心评估（blinded independent central review, BICR）评估的cORR为59.8%（58/97），对于基线伴有脑转移的患者，cORR为48.4%，研究共纳入30种EGFR ex20ins突变亚型，其中71例患者插入突变发生在Loop环近端，ORR达62%；24例患者插入突变位置发生在Loop环远端，ORR达50%，而其DOR、中位PFS及中位OS数据目前尚不成熟，需要更长时间进行随访。安全性方面，最常见的≥3级AEs为腹泻（7.3%）、皮疹（2.8%）和血液磷酸肌酸激酶（creatine phosphokinase, CPK）升高（13.5%），20.2%的患者因AEs导致减量，7.9%因AEs导致停药，33.1%因AEs导致药物中断。此外，舒沃替尼目前正在进行一项开放标签、随机、多中心III期研究（CTR20223235）以评估舒沃替尼对比含铂双化疗在一线治疗局部进展或转移性EGFR ex20ins突变NSCLC中的疗效及安全性。

Amivantamab（JNJ-372）为全人源EGFR-间质表皮转化因子（mesenchymal-epithelial transition, MET）双特异性抗体。已于2021年5月在美国获批，用于治疗含铂化疗经治的EGFR ex20ins突变NSCLC，目前尚未在国内获批。基于CHRYSALIS多中心、开放性、多队列、I期研究（NCT02609776）的结果^[48]^，截止2022年9月12日，经过19.2个月的随访，共114例EGFR ex20ins突变晚期NSCLC成人患者纳入有效性分析。研究结果显示，基于INV评估的cORR为37%（42/114），基于INV评估的DOR为12.5个月，中位PFS为6.9个月，中位OS为23个月。既往接受过免疫治疗患者的ORR为42%（21/50），既往接受过EGFR-TKIs治疗患者的ORR为52.2%（12/23）。Amivantamab作为靶向EGFR和MET通路的双特异性抗体，其最常见的TRAEs为皮疹（89%），其次为输液反应（67%）、甲沟炎（58%）、痤疮样皮炎（43%）等，除此之外，还会引起MET相关AEs，如低蛋白血症（39%）、周围性水肿（27%）。其中≥3级AEs占16%，18%的患者因AEs导致减量，7%因AEs导致停药，29%因AEs导致剂量中断。其一线研究PAPILLON（NCT04538664）^[49]^是一项国际多中心、随机、开放性、III期临床研究，目前正在开展中，将评估Amivantamab联合卡铂与培美曲塞对比卡铂与培美曲塞治疗的疗效及安全性。

伏美替尼（Furmonertinib, AST2818) 作为中国原发研究的第三代EGFR-TKIs，其IB期研究FAVOUR（NCT04858958）^[50]^队列1也纳入了10例EGFR ex20ins突变NSCLC初治患者，截止2021年6月试验数据显示伏美替尼治疗EGFR突变NSCLC的IRC评估的cORR为60%，INV评估的cORR为70%，初步显示出良好的抗肿瘤活性和耐受性，期待更大样本量的研究进一步验证其疗效。安全性方面，最常见的TRAEs有腹泻、甲沟炎、皮肤皲裂（各占30%），未观察到≥3级的TRAEs，未发生减量和停药。有关该药的一项II期、多中心、开放标签研究（CTR20221165）也正在国内开展，旨在评价其在EGFR ex20ins突变的局部晚期或转移性NSCLC患者中的疗效及安全性，研究剂量为每日空腹口服240 mg，主要终点为BICR评估的cORR，目前研究状态为招募中。一项评估伏美替尼对比含铂化疗一线治疗EGFR ex20ins突变NSCLC的III期、随机、多中心、开放标签研究FURMO-004（CTR20231409/NCT05607550）^[51]^正在进行中，主要终点为BICR评估的PFS。

BEBT-109是国内自研的一种高活性的泛突变型 EGFR抑制剂，是第三代EGFR-TKIs奥希替尼化学结构类创新药物，目前也在EGFR ex20ins突变局部晚期或转移性NSCLC患者中进行多中心、开放的II期临床试验（CTR20213409），研究剂量为每日两次，每天早、晚餐前口服，每次120 mg，主要终点为BICR评估的cORR，目前研究状态为招募中。

正在EGFR ex20ins突变NSCLC进行探索的新型化合物还包括JMT-101、BLU-451和YK-029A等，期待未来有更多的证据出现，以期更好地改善该类患者的生存预后。以上研究具体信息列于表4。

**表4:** 针对EGFR ex20ins突变NSCLC一线及后线治疗的新型化合物临床研究

研究	一线/后线	分期	治疗方案	主要终点	目前状态	不良反应/安全性数据
EXCLAIM-2^[45]^ （NCT04129502）	一线	III期	莫博赛替尼 vs 含铂化疗（培美曲塞+顺铂或卡铂继以培美曲塞维持）	IRC评估的PFS	进行中	尚未披露
CTR20223235	一线	III期	舒沃替尼 vs 含铂化疗（培美曲塞+卡铂）	BICR评估的PFS	进行中	尚未披露
PAPILLON^[49]^ （NCT04538664）	一线	III期	Amivantamab联合卡铂和培美曲塞 vs 卡铂与培美曲塞治疗	BICR评估的PFS	进行中	尚未披露
FAVOUR^[50]^ （NCT04858958）	一线	IB期	伏美替尼	IRC评估的cORR	进行中，截止2021年6月数据：IRC评估的cORR为60%，INV评估的cORR为70%	最常见的AEs: 腹泻占30%，甲沟炎占30%，皮肤皲裂 占30%；未观察到≥3级的AEs；未发生AEs导致减量 和停药
FURMO-004^[51]^ （CTR20231409） （NCT05607550）	一线	III期	伏美替尼 vs 含铂化疗	BICR评估的PFS	进行中	尚未披露
WU KONG 6 （CTR20211009）	后线	II期	舒沃替尼	BICR评估的cORR	进行中，截止2022年7月31日数据：BICR评估的cORR为59.8%	最常见的AEs: 腹泻占7.3%，皮疹占2.8%，血液CPK升高占13.5%；AEs导致减量 占20.2%，导致停药占7.9%，导致剂量中断占33.1%
CHRYSALIS （NCT02609776）队列4	后线	I期	Amivantamab	INV评估的cORR	进行中，截止2022年9月12日数据：INV评估的cORR为37%（95%CI: 28%-46%），mDOR为12.5个月（95%CI: 6.9-19.3），mPFS为6.9个月（95%CI: 5.6-8.8），mOS为23个月（95%CI: 18.5-29.5）	最常见的AEs: 皮疹占89%，输液反应占67%，甲沟炎 占58%，痤疮样皮炎占43%，低蛋白血症占39%，周围性水肿占27%，≥3级的AEs占16%；AEs导致减量占18%，导致停药占7%，导致剂量 中断占29%
CTR20221165	后线	II期	伏美替尼	BICR评估的cORR	进行中	尚未披露
CTR20213409	后线	II期	BEBT-109	BICR评估的cORR	进行中	尚未披露
NCT04448379	后线	I期	JMT101联合阿法替尼或奥希替尼	安全性和耐受性	进行中	尚未披露
BLU-451-1101 （NCT05241873）	后线	I期/II期	BLU-451	I期：MTD、RP2D、安全性和耐受性； II期：ORR	I期进行中	尚未披露

BICR：盲态独立中心评估；AEs：不良事件；CPK：磷酸肌酸激酶；mDOR：中位缓解持续时间；MTD：最大耐受剂量；RP2D：推荐的II期剂量；cORR：确认的ORR。

## 结语与展望

随着NSCLC精准诊疗的发展，EGFR ex20ins突变作为罕见突变亚型逐渐被关注，但其异质性高，不同插入位点亚型临床获益不同，预后极差，对传统治疗原发耐药，常规PCR漏检率高，因此，在临床诊疗过程中更应该引起高度重视。本共识专家组通过国内外文献及临床数据的参考，并且结合专家自身临床经验，形成EGFR ex20ins突变NSCLC临床规范化诊疗专家共识，共识指出：（1）在疾病认知方面，应当提高对EGFR ex20ins突变靶点及其疾病临床特征的认知；（2）在检测方面，EGFR ex20ins突变常规PCR漏检率高达50%，推荐有条件的患者优先选择NGS检测，同时推动上下级医院联动、院内临理协作，以提高检出率，为患者制定最大获益的精准治疗策略；（3）在治疗方面，目前国内尚无一线治疗EGFR ex20ins突变NSCLC靶向药物获批，当前可参考局部晚期或转移性无驱动基因的NSCLC的一线治疗，而对于无法耐受或拒绝化疗/免疫治疗或PS评分较差等特殊的患者，一线可选择莫博赛替尼。EGFR ex20ins突变NSCLC后线治疗中，莫博赛替尼作为国内首个且唯一获批口服靶向药，填补了临床空白，开启了该领域靶向治疗的新纪元，为含铂化疗经治的EGFR ex20ins突变NSCLC患者带来长期生存获益，推荐莫博赛替尼作为这类患者的治疗优选；（4）无论是对于EGFR ex20ins突变NSCLC一线还是后线治疗，多种新型靶向药物的临床试验正在如火如荼的开展中，包括莫博赛替尼、Amivantamab、舒沃替尼、伏美替尼、FWD1509 MsOH、BEBT-109胶囊、JMT-101、BLU-451和YK-029A等，临床医生可持续关注相关循证医学证据的产出，本专家共识也会根据临床数据、新药的获批、检测技术的发展等定期更新，进而推动EGFR ex20ins突变NSCLC规范化诊疗的发展，使更多肺癌患者获益。

**Table:** 

参与本共识的专家组成员	
专家组组长	
周彩存	同济大学附属上海市肺科医院
专家组成员（按姓氏汉语拼音字母排序）	
褚倩	华中科技大学同济医学院附属同济医院
崔久嵬	吉林大学第一医院
黄媚娟	四川大学华西医院
何志勇	福建省肿瘤医院
李琳	北京医院
马智勇	郑州大学附属肿瘤医院/河南省肿瘤医院
潘跃银	中国科学技术大学附属第一医院
任胜祥	同济大学附属上海市肺科医院
史美祺	江苏省肿瘤医院
沈毅弘	浙江大学医学院附属第一医院
宋启斌	武汉大学人民医院
王佳蕾	复旦大学附属肿瘤医院
王立峰	南京大学医学院附属南京鼓楼医院
王启鸣	郑州大学附属肿瘤医院/河南省肿瘤医院
王哲海	山东第一医科大学附属肿瘤医院
邬麟	湖南省肿瘤医院
赵明芳	中国医科大学附属第一医院
